# Case report: Early carotid stent shortening in patient with radiation-induced carotid stenosis

**DOI:** 10.3389/fneur.2023.1184210

**Published:** 2023-05-09

**Authors:** Woochan Choi, Yang-Ha Hwang, Yong-Won Kim

**Affiliations:** ^1^Department of Neurology, Kyungpook National University Hospital, Daegu, Republic of Korea; ^2^Department of Neurology, School of Medicine, Kyungpook National University, Daegu, Republic of Korea

**Keywords:** radiation-induced carotid stenosis, carotid stent, carotid artery stenosis, stent shortening, closed cell stent

## Abstract

Carotid artery stenting (CAS) for carotid stenosis has been widely used as an alternative treatment in patients not eligible for surgery. The shortening of a carotid stent rarely occurs. We report a case of early shortening of CAS in a patient with radiation-induced carotid stenosis and discuss the potential pathophysiology and strategies for prevention. This case presents a 67-year-old man who underwent radiotherapy for oral cavity squamous cell carcinoma 7 years ago and subsequently developed severe stenosis in the left proximal internal carotid artery. The patient underwent CAS for symptomatic severe carotid stenosis. Follow-up CT angiography revealed shortening of the carotid stent, and additional carotid stenting was performed. We speculate that the possible mechanism of early complication of CAS could be slippage and shortening of the stent due to weak anchoring between the stent strut and the fibrotic arterial wall in radiation-induced carotid stenosis.

## Introduction

Cervical radiation therapy for head and neck malignancies may cause extracranial carotid stenosis and consequently lead to a higher risk of cerebrovascular events such as transient ischemic attack and stroke. Radiation-induced carotid stenosis (RICS) has a reported incidence of up to ~20% in long-term follow-up studies (1). Carotid endarterectomy for RICS is considered a high-risk procedure in comparison with carotid artery stenting (CAS) due to arterial wall fibrosis, difficult wound healing, and an increased risk of cranial nerve injuries (2). Accordingly, CAS has been used more frequently in RICS patients. The shortening of a carotid stent rarely occurs. We report a case of early shortening of CAS in an RICS patient and discuss the potential pathophysiology and strategies for prevention.

## Case presentation

A 67-year-old man presented to the emergency department with complaints of right hemiparesis following improvement after onset. On arrival, he presented with right-hand clumsiness. His National Institutes of Health Stroke Scale (NIHSS) score was 1. He had been treated with radiotherapy for oral cavity squamous cell carcinoma 7 years ago and then underwent wide excision with left marginal mandibulectomy and reconstruction. He had no vascular risk factors, such as hypertension, diabetes, or hyperlipidemia, except smoking.

**Figure 1:**
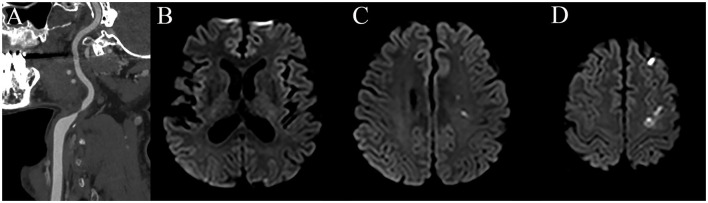
CT angiography shows severe stenosis in the left proximal ICA **(A)** and diffusion-weighted imaging reveals multiple scattered acute infarctions in the left hemisphere **(B–D)**.

Computerized tomography angiography (CTA) revealed severe stenosis of the left proximal internal carotid artery (ICA; Figure 1A). Diffusion-weighted imaging showed multiple scattered cortical infarctions along the left cerebral hemisphere (Figures 1B–D). The patient started aspirin 100 mg, clopidogrel 75 mg, and atorvastatin 40 mg.

**Figure 2:**
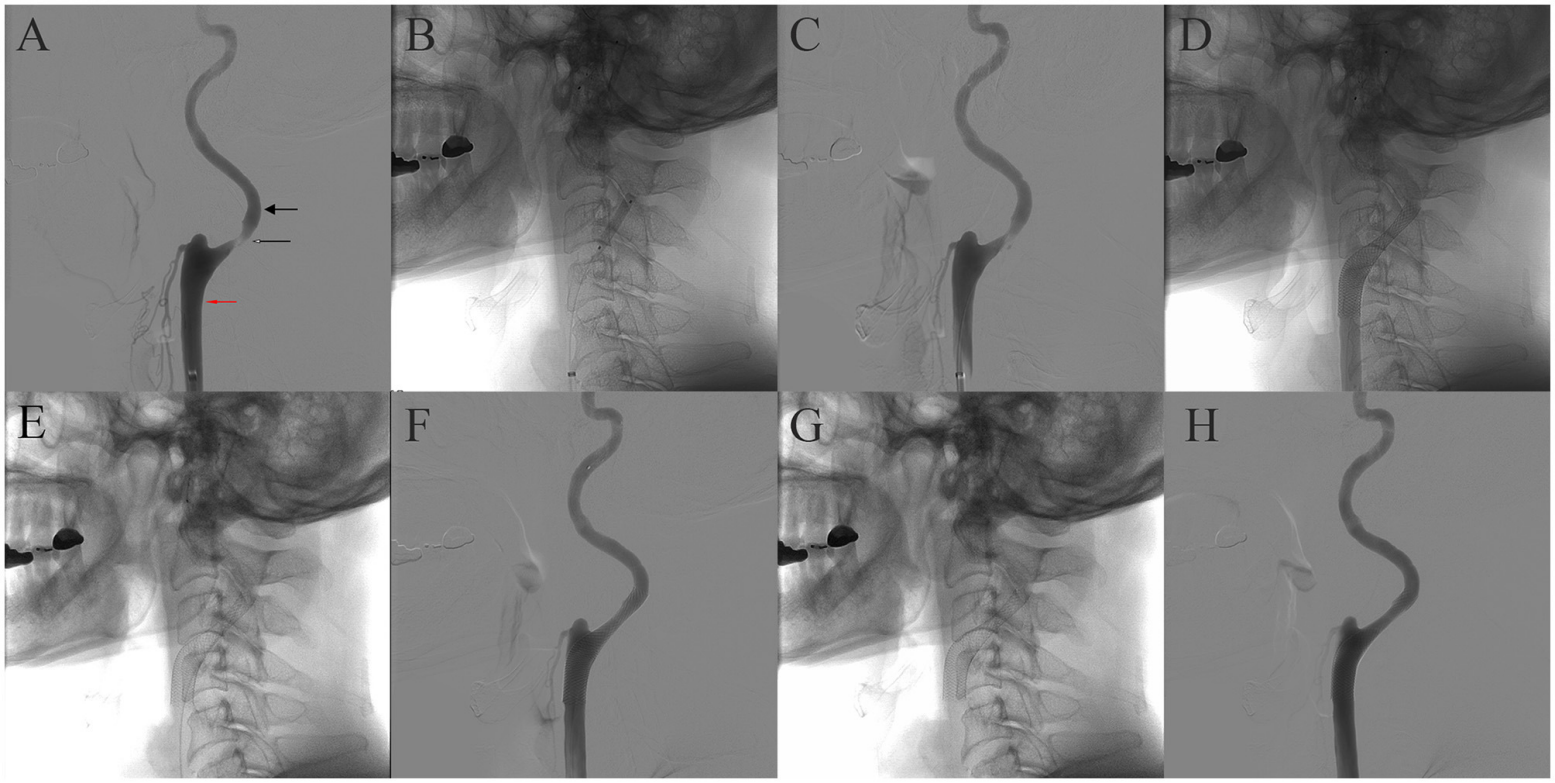
Sequential images of the carotid stenting. Cerebral angiography revealed ~87% stenosis of the left ICA according to NASCET criteria. The diameter of the stenotic segment was 0.56 mm (thin arrow), and the diameter of the distal ICA was 4.41 mm (thick arrow), but the diameter of the distal common carotid artery was 7.63 mm (red arrow) **(A)**. **(B)** Balloon angioplasty before carotid stenting. **(C)** Angiography after balloon angioplasty. **(D)** Carotid Wallstent deployment. **(E)** Native fluoroscopy after poststent balloon angioplasty. **(F)** Angiography after post-stent balloon angioplasty. **(G, H)** Native fluoroscopy and final angiography.

Then, 5 days after starting the medication, trans-femoral cerebral angiography confirmed 87% stenosis in the left proximal ICA using NASCET criteria (Figure 2A). Due to symptomatic severe carotid stenosis, CAS was performed under local anesthesia. A 6-Fr shuttle sheath (Cook Medical, Bloomington, IN, USA) was positioned from the right femoral artery to the left common carotid artery. Then, a microguidewire was navigated into the ICA under road map guidance, and an embolic protection device (EPD, SpiderFx 5.0 mm; Medtronic, Minneapolis, MN, USA) was deployed in the pre-petrous segment of the ICA. Prior to CAS, balloon angioplasty was performed using a 4 × 20 mm angioplasty balloon (Submarine Rapido, Invatec, Italy; Figures 2B, C). Subsequently, an 8 × 36 mm Carotid Wallstent (Boston Scientific, Natick, MA, USA) was deployed across the stenotic lesion (Figure 2D). We performed additional balloon angioplasty after carotid stenting for stent apposition (Figures 2E, F). After the careful withdrawal of the EPD, a carotid angiogram demonstrated successful stent implantation without residual stenosis (Figures 2G, H).

**Figure 3:**
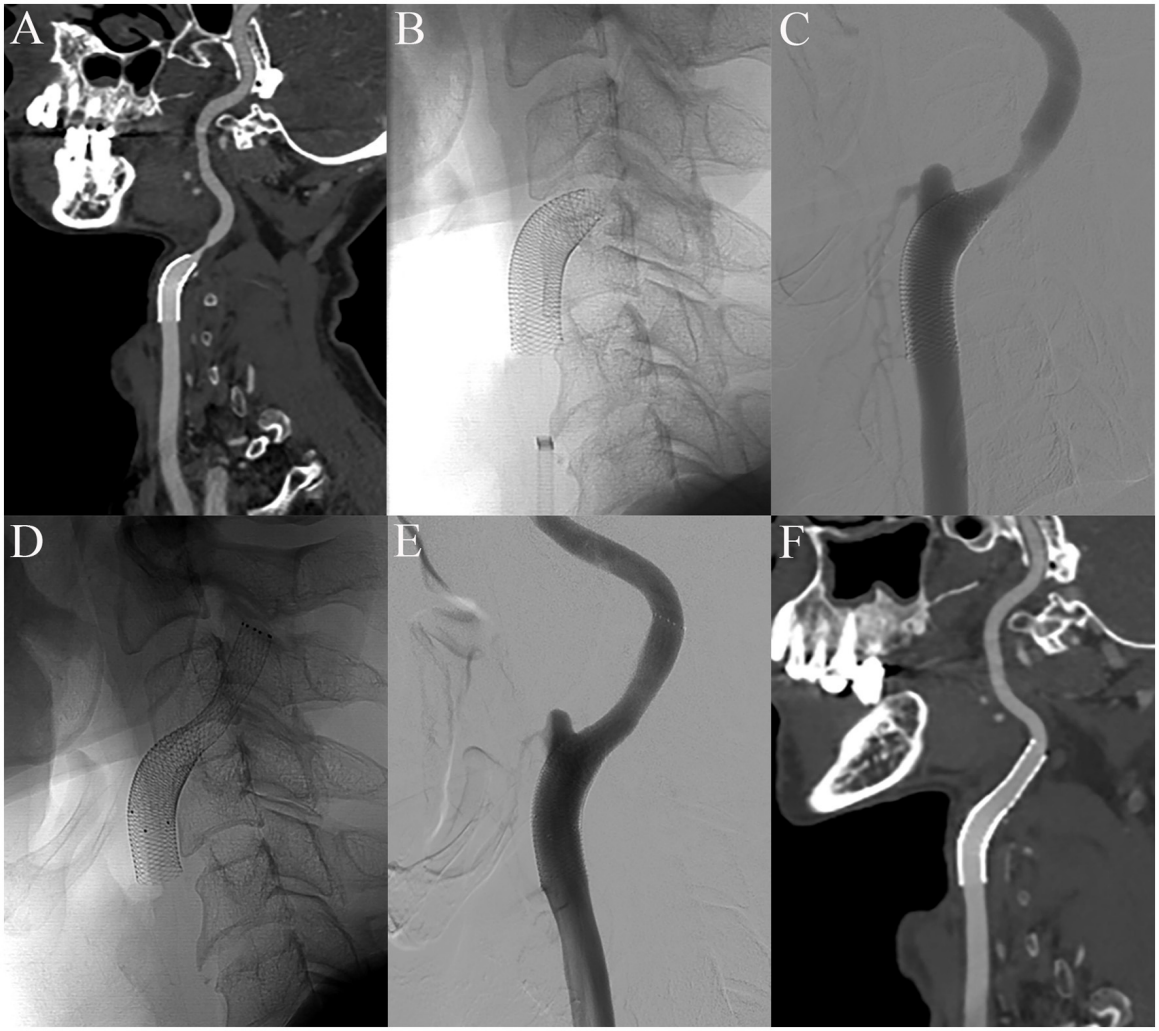
The day after CAS, early shortening of the CAS and distal stenosis are demonstrated in CTA and the cerebral angiogram **(A–C)**. After additional carotid stenting, the final angiography showed successful expansion and implantation of the carotid stent **(D, E)**. There was no shortening and malposition of the CAS 1 month after additional carotid stenting **(F)**.

Follow-up CTA and cerebral angiography were performed the day after CAS showed downward shortening of the carotid stent and exposed stenotic area (Figures 3A–C). Therefore, we planned additional CAS. A 6-Fr shuttle sheath was positioned from the left femoral artery to the left common carotid artery. A microguidewire was gently navigated into the ICA across the carotid stent and stenotic segment, and an EPD (SpiderFx 5.0 mm) was deployed in the pre-petrous segment of the ICA. Balloon angioplasty was performed using a 5 × 20 mm balloon (Submarine Rapido) at the stenotic segment. Subsequently, an 8 × 40 mm Protégé RX stent (Medtronic, Minneapolis, MN, USA) was deployed across the lesion including the more distal segment of the ICA. Successful expansion and implantation were confirmed by the final angiogram (Figures 3D, E). A 1-month follow-up CTA showed successful implantation of the stent without shortening and malposition (Figure 3F).

## Discussion

Early shortening of CAS is a rare and unexpected complication. There were only a few case reports related to carotid stent shortening in patients with severe carotid stenosis (3–8). Most cases reported delayed onset carotid stent shortening except for one case report (3). Previous reports have suggested high-grade stenosis degree and a closed-cell type stent as potential risk factors (3–6). Carotid stenosis related to atherosclerosis is associated with local intimal thickening of the arterial wall, plaque formation, and growth (9). Therefore, we can speculate that the arterial wall in high-grade carotid stenosis can be more rigid than a normal arterial wall. Thus, a severe stenotic segment in carotid arteries may cause a squeezing force toward the lesser stenotic area, which could lead to slipping and migration away from the initial deployment of the carotid stent. This phenomenon has been known as the “watermelon seeding” effect (8). This phenomenon can be also commonly seen during balloon angioplasty. Thus, if balloon slippage occurs during balloon dilatation, we should consider the risk of shortening and migration of CAS.

The type and size of the carotid stent can be potential risk factors. The Carotid Wallstent is braided from continuous filament, self-expandable closed-cell type, and is characterized by a small free cell area between struts, which can cover an increased area and have additional stabilization (10). However, this design is less flexible and may develop kinks and incomplete expansion. As a Carotid Wallstent has monofilament wires with a braided design, the length of the stent can change according to the diameter of the artery of the landing zone. According to an *in vitro* model study, Carotid Wallstent showed a 22% length reduction following stent expansion (11). Therefore, when the stent slips out from the stenosis to a wide area, rapid shortening of the carotid stent may occur. To overcome this complication, an open-cell design stent can be an alternative. Because the open-cell stent has a segmented design, it is flexible and provides better stent apposition, especially in angulated vessels or tortuous anatomy (10). Additionally, the size of the carotid stent should be considered. Park et al. also reported two cases of early carotid stent shortening (3). They suggested a smaller diameter of the stent than the common carotid artery (CCA) as a possible cause. In another case, delayed carotid stenting shortening by this mechanism was reported (4). However, we used a slightly larger diameter of the carotid stent than the CCA and believe that there was no mismatch of diameters between the CCA (7.63 × 7.25 mm) and the carotid stent (8 mm).

However, the carotid stenosis in this case was associated with radiation. RICS has a complex mechanism that includes atherosclerosis (12). Radiation exposure can destroy the internal elastic layer, fray elastic fibers, degenerate smooth muscle, and cause extensive fibrosis of the vessel wall. It also induces occlusion of the vasa vasorum causing fibrosis of the adventitia. Therefore, the arterial wall can become stiff by fibrosis in all three layers of the arterial wall. Moreover, degeneration of smooth muscle and fraying of elastic fibers can affect the contractility of the arterial wall, which can mean loss of elastic recoil (12, 13). We can easily notice these characteristics during balloon angioplasty. Compared to atherosclerotic carotid stenosis, the balloon can be fully dilated even with low inflation pressure during balloon angioplasty in radiation-induced carotid stenosis. The watermelon seeding phenomenon rarely occurs, and it is difficult to predict stent slippage and shortening prior to CAS. Furthermore, decreased elastic recoil of the arterial wall also affects incomplete stent apposition and anchoring of the stent strut. Therefore, we can speculate that the possible mechanism of early shortening of CAS, in this case, could be slippage and shortening of the stent due to decreased elastic recoil of the arterial wall rather than a squeezing force by high-grade stenosis.

Furthermore, the location of the carotid stent should also be taken into consideration. In this case, the Carotid Wallstent covered the entire stenotic segment but did not cover much of the distal non-stenotic segment due to the downward displacement of the guide catheter during stent delivery. Consequently, the carotid stent was deployed in proximal ICA, where it could be delivered without kickback of the guide catheter. It is important to note that the distal segment of the Carotid Wallstent is the weakest part due to its braided design. Therefore, carotid stent shortening could have been avoided if it had been placed more distally so that the severe stenotic segment with decreased elasticity was in the middle of the stent, thus avoiding the weak distal part of the stent.

In our case, stent shortening occurred within 24 h after the procedure. It could be associated with decreased elastic recoil of the arterial wall due to radiation and the closed-cell designed stent. Therefore, the cause of carotid stenosis and the size and type of carotid stent should be considered to prevent this complication. In radiation-induced carotid stenosis, the use of an open-cell type or slightly larger or longer stent may be a possible alternative. Short-term follow-up evaluation might be also important to detect unexpected complications.

## Data availability statement

The original contributions presented in the study are included in the article/supplementary material, further inquiries can be directed to the corresponding author.

## Ethics statement

The studies involving human participants were reviewed and approved by Kyungpook National University Hospital Institutional Review Board. Written informed consent was obtained from the patient for the publication of any potentially identifiable images or data included in this article.

## Author contributions

WC interpreted the data and wrote the manuscript. Y-HH revised the manuscript for intellectual content. Y-WK designed and conceptualized the study, interpreted the data, and drafted the manuscript for intellectual content. All authors have read and approved the manuscript.
